# Decoy Receptors in Autoimmunity: Molecular Guardians and Pathogenic Players in Immune Dysregulation

**DOI:** 10.1155/mi/7430042

**Published:** 2026-01-11

**Authors:** Hadiseh Farahani, Parviz Kokhaei, Ali Ganji, Ghasem Mosayebi, Ali Ghazavi

**Affiliations:** ^1^ Department of Immunology, School of Medicine, Arak University of Medical Sciences, Arak, Iran, arakmu.ac.ir; ^2^ Molecular and Medicine Research Center, Arak University of Medical Sciences, Arak, Iran, arakmu.ac.ir; ^3^ Traditional and Complementary Medicine Research Center (TCMRC), Arak University of Medical Sciences, Arak, Iran, arakmu.ac.ir; ^4^ Infectious Diseases Research Center (IDRC), Arak University of Medical Sciences, Arak, Iran, arakmu.ac.ir

**Keywords:** autoimmune disease, decoy receptor, immune regulation, inflammation

## Abstract

Autoimmune disorders encompass a varied range of diseases in which the immune system mistakenly targets and attacks the body’s own tissues. The causes of the conditions are unknown. It is presumed that various genetic, environmental, and immune factors all play a part. Nowadays, therapies concentrate mainly on anti‐inflammatory agents with immunosuppressant medications. New research highlights the central role of decoy receptors (DcRs) in regulating the immune system. DcRs are molecular traps for cytokines and other signaling molecules, preventing them from binding to functional receptors and influencing inflammatory processes. Their activity is context‐dependent, shifting the balance between protective and pathogenic responses, and DcR dysregulation has been implicated in the development of autoimmune diseases. Understanding DcR function is critical for the design of potential therapeutic interventions. DcR mechanisms are reviewed here with emphasis on structural and disease‐specific functions. Targeting DcRs is a promising strategy to reconstitute immune homeostasis. Understanding the dual regulatory functions and context‐dependent mechanisms is critical for designing new therapies that reduce autoimmune pathogenesis without compromising host defense mechanisms.

## 1. Introduction

Autoimmune diseases are a range of conditions resulting from the body’s abnormal immune response against its own cells and tissues [1]. This misguided response triggers ongoing inflammation and damage to tissues, potentially resulting in a variety of health issues. The precise causes of autoimmune diseases remain unclear; however, it is believed that genetic, environmental, and immunological factors contribute to their onset. Treatment for autoimmune diseases typically involves immunosuppressive drugs targeting specific molecules involved in the inflammatory response [1]. A deeper understanding of immune regulators is important for developing targeted strategies that restore immune balance without compromising host defense [2]. Among these regulators, decoy receptors (DcRs) have emerged as pivotal molecules capable of regulating inflammation by sequestering cytokines, chemokines, and apoptotic ligands [3].

DcRs are variants that can recognize specific inflammatory cytokines with varying degrees of affinity and specificity. However, they are structurally unable to initiate signaling, or they may signal through pathways distinct from those used by the canonical receptors with which they share ligands. These receptors function as negative regulators by either trapping agonists and signaling components or serving as scavenger receptors that guide cytokines into intracellular compartments for degradation [4, 5]. So, they modulate critical pathways for immune activation, apoptosis, and cytokine amplification [5]. However, their role is context‐dependent; they can either prevent or promote the development of autoimmune diseases, underscoring the complexity of DcRs [6, 7]. The importance of DcRs in autoimmune diseases is becoming a focal point in research. These receptors serve as decoys for cytokines and various signaling molecules and are crucial to the progression of autoimmune conditions. By attaching to these molecules, DcRs inhibit interactions between immune cells and the host [5]. When this regulation fails, it can trigger an excessive immune response, leading to tissue damage [8]. Clarifying the role of DcRs in autoimmune diseases can lead to the development of new and improved treatments. This article will explore the current research on DcRs in autoimmune diseases, discussing their structural and mechanisms of action.

## 2. DcRs in Autoimmune Diseases

DcRs can regulate immune responses by interfering with the binding of proinflammatory cytokines to their receptors on immune cells. This can prevent the activation of signaling pathways responsible for causing inflammation and harming tissues [3, 5]. Several DcRs have been identified that play a role in the pathogenesis of autoimmune diseases such as DcR3/TR6, interleukin‐1 receptor 2 (IL‐1RII), tumor necrosis factor (TNF)‐related apoptosis‐inducing ligand receptors (TRAIL receptors), soluble TNF receptors (sTNFRs), IL‐18 binding protein (IL‐18BP), vascular endothelial growth factor receptor‐1 (VEGFR‐1), and atypical chemokine receptors (ACKRs) [9]. In the following, we will explain these DcRs in the context of autoimmune diseases.

### 2.1. DcR3/TR6

**Figure 1:**
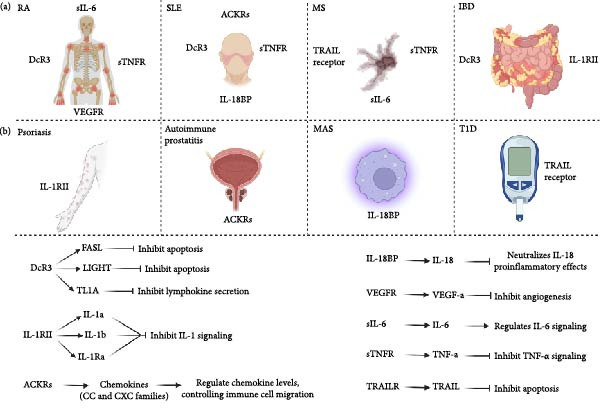
Mechanisms of decoy receptors in autoimmune diseases. (A) Different autoimmune diseases with their associated key decoy receptors. (B) Decoy receptors modulate immune homeostasis by sequestering proinflammatory ligands, including FasL, LIGHT, TL1A, IL‐1a, IL‐1b, IL‐1Ra, chemokines, IL‐18, VEGF‐a, IL‐6, TNF‐a, and TRAIL, thereby regulating apoptosis, cytokine signaling, angiogenesis, and immune cell migration, playing a protective or pathogenic role in autoimmune diseases.

DcR3 is a 33 kDa glycosylated soluble protein and a member of the tumor necrosis factor receptor superfamily (TNFRSF). Unlike most TNFRSF members, DcR3 lacks a transmembrane domain [10]. Operating as a DcR, DcR3 binds to and hinders three specific ligands: Fas ligand (FasL/TNFSF6/CD95L), LIGHT (CD258/TNFSF14), and TNF‐like molecule 1A (TL1A/TNFSF15) (Figure 1) [11].

DcR3 interacts with FasL, blocking the connection between Fas and FasL and consequently preventing apoptosis induced by FasL [12]. Furthermore, DcR3 can attach to LIGHT, offering protection against apoptosis triggered by LIGHT [13]. It also reduces the secretion of lymphokines that TL1A enhances [12, 14].

DcR3 interacts with specific ligands and is linked to autoimmune diseases like rheumatoid arthritis (RA), systemic lupus erythematosus (SLE), and inflammatory bowel disease (IBD) [15].

In SLE, DcR3 is overexpressed in peripheral blood mononuclear cells (PBMCs), and this increased expression is associated with disease activity and severity [16]. Overproduction and increased expression of DcR3 can prevent Fas from binding to FasL, thereby diminishing activation‐induced cell death (AICD) [12]. DcR3 has also been demonstrated to enhance the longevity of autoreactive B cells and Th17 cells, which are crucial in the development of SLE [17]. By binding to TL1A, DcR3 prevents the formation of the complex between TL1A and its receptor, DR3, thereby inhibiting T‐cell activation and the production of proinflammatory cytokines [17].

In RA patients, DcR3 can be detected in the chondrocytes of both osteoarthritis (OA) patients and normal individuals [18]. DcR3 levels in the synovial fluids and serum of RA patients are significantly higher than in OA patients. Furthermore, DcR3 mRNA and protein are found in fibroblast‐like synoviocytes (FLS) in RA [17] but not in OA patients [19]; however, this is not apply to OA patients [20]. IL‐23 induces DcR3 mRNA expression in RA‐FLS, which is critical in activating IL‐17 and IL‐6‐mediated inflammatory disease [21].

DcR3 prevents TL1A/DR3 binding, implying a protective role during chronic synovial inflammation [20]. It was reported that DcR3 could bind to TL1A expressed on FLS from RA patients and inhibit their proliferation induced by proinflammatory cytokines [22].

Studies have demonstrated that DcR3 can inhibit FasL‐mediated apoptosis of FLS, leading to increased proliferation and survival of these cells [6]. Additionally, DcR3 has been shown to promote Th17 differentiation, which plays a critical role in RA pathogenesis [23].

DcR3 is significantly elevated in individuals with IBD [24, 25]. Furthermore, the serum concentration of DcR3 is elevated in patients diagnosed with primary Sjögren’s syndrome (pSS). It could prevent apoptosis of activated T cells and glandular epithelial cells [26]. DcR3 was overexpressed in both actively inflamed and inactive regions of the ileal epithelium in patients with Crohn’s disease (CD) [27]. People with CD had higher DcR3 expression levels than healthy people. In intestinal epithelial cells, DcR3 expression is promoted by TNF‐α [28]. Research has established a connection between the activation of nuclear factor‐kappa B (NF‐kB) and an increase in DcR3 expression. As a result, DcR3 inhibits apoptosis triggered by FASL in T cells located in the lamina propria and in intestinal epithelial cells. DcR3 may help reduce inflammation in CD by decreasing FASL‐induced cell death in both immune and epithelial cells, while also promoting the activation of NF‐kB [27, 29]. DcR3 has the potential to mitigate the proinflammatory effects of TL1A. Consequently, increased DcR3 expression during intestinal inflammation may serve a compensatory and protective role [29, 30].

### 2.2. IL‐1 Receptor Type II (IL‐1RII)

IL‐1RII is a DcR for IL‐1. It is also known as CD121b. It binds IL‐1α, IL‐1β, and IL‐1 receptor antagonist (IL‐1Ra) and inhibits their binding to conventional receptors (Figure 1) [5].

The IL‐1 cytokine family, particularly IL‐1α and IL‐1β, is known for its significant involvement in the pathophysiology of various autoimmune diseases [31]. These cytokines facilitate a cascade of inflammatory responses by binding to IL‐1RI, activating NF‐κB and mitogen‐activated protein kinases (MAPKs) [32]. Conversely, IL‐1RII, lacking an intracellular signaling domain, fails to convey these inflammatory signals. Instead, it traps IL‐1, reducing its availability to IL‐1RI and inhibiting the inflammatory response. IL‐1RII has been implicated in the pathogenesis of autoimmune diseases such as RA, IBD, and psoriasis [30].

Studies have shown increased concentrations of IL‐1RII in synovial fluid and plasma from RA patients [33]. Levels of IL‐1RII negatively correlate with disease severity, implicating IL‐1RII as a natural antagonist of IL‐1‐driven joint destruction [33, 34].

By binding IL‐1β, IL‐1RII reduces IL‐1β’s proinflammatory effects in the gut, which is crucial for managing the intestinal inflammation characteristic of IBD [35]. In IBD patients, IL‐1RII levels decrease while IL‐1R1 levels increase. The balance between proinflammatory IL‐1RI and anti‐inflammatory IL‐1RII is disrupted in patients with IBD, resulting in a poorer prognosis [36].

Psoriasis is an autoimmune skin disease characterized by hyperproliferation of epidermal keratinocytes and significant immune cell infiltration. Research indicates that IL‐1 is vital for the formation and maintenance of psoriatic plaques [37]. IL‐1RII is strongly upregulated in human psoriatic skin [38] and decreases significantly after treatment, demonstrating its role in reducing inflammation by inhibiting the IL‐1 pathway [37].

Since IL‐1RII is expressed by both Th17 cells and regulatory T cells (Tregs), it has been proposed that IL‐1RII contributes to the plasticity of these cells. Specifically, it may participate in the trans‐differentiation of Th17 cells into Tregs, thereby contributing to the resolve of inflammation. IL‐1β is an important co‐signal for human Th17 differentiation and expansion. IL‐1RII binds IL‐1 but lacks signaling capacity, thereby decreasing IL‐1 bioavailability and IL‐1R1 signaling. This leads to a lower activation of Th17 cells. Recent reviews and experimental studies indicate that IL‐1RII limits IL‐1’s effects on Th17 cells and can be induced in regulatory settings, linking the induction of DcRs to the regulation of IL‐17 by Treg cells [39–41].

### 2.3. TRAIL Receptors

TRAIL is a type II transmembrane protein in the TNF superfamily [42]. TRAIL interacts with five different receptors: it binds to two death receptors (DRs), TRAIL‐R1 (DR4) and TRAIL‐R2 (DR5), which promote apoptosis. Additionally, it binds to two DcRs, TRAIL‐R3 (DcR1) and TRAIL‐R4 (DcR2), that act as dominant‐negative inhibitors of TRAIL‐mediated apoptosis, and soluble receptor osteoprotegerin (OPG) [43]. DcR1 and DcR2 serve as DcRs and cannot induce signaling because they lack the intracellular death domain; they can compete with DR4 and DR5 for TRAIL binding, thereby inhibiting TRAIL‐induced apoptosis (Figure 1) [44, 45].

TRAIL induces cell apoptosis by binding to its DRs [46, 47]. Additionally, it inhibits the activation of autoreactive T cells and hinders the progression of autoimmune diseases through a pathway that does not involve apoptosis, utilizing TRAIL‐R signaling [42]. In RA, a lack of TRAIL or its blockade leads to increased joint inflammation and heightened disease severity [48, 49]. In contrast, TRAIL administration reduces joint inflammation and limits synovial lymphocyte infiltration in the RA model [50]. In another study, the increased expression of DCR1 and DCR2 in RA was positively correlated with disease severity [51].

In patients with multiple sclerosis (MS), high DcR1 expression was observed in antigen‐specific T cells [52]. Additionally, these antigen‐specific T‐cell clones showed resistance to TRAIL‐induced apoptosis. Antigen‐specific T‐cell clones may enhance survival following DcR1 upregulation [46].

In IBD, TRAIL expression in intestinal epithelial cells is decreased [53]. However, TRAIL levels are significantly elevated in mononuclear cells, particularly in cases of severe ulcerative colitis (UC) and CD [54]. Studies indicate that, in conditions characterized by inflammation, TRAIL is a strong trigger for programmed cell death in intestinal epithelial cells [29, 54].

Type 1 diabetes (T1D) is an autoimmune disease that targets and destroys pancreatic β‐cells [55]. Studies revealed increased DcR1 expression in T1D [43]. When INS‐1 cells, an insulin‐secreting pancreatic β‐cell line, were treated with TRAIL, no apoptosis of the islet β‐cells occurred; NF‐kB was activated, and DcR1 expression increased [43]. Suppression or removal of TRAIL activity has been shown to exacerbate the pathophysiology of T1D [56], likely through NF‐kB activation and heightened expression of DcRs [43].

### 2.4. sTNFRs

TNF‐α plays a vital role in the immune system. When TNF‐α attaches to TNFRs, it triggers inflammatory and stress response pathways [57]. There are two primary TNF‐α receptors: type 1 (TNFR1) and type 2 (TNFR2) [5]. TNFRs are bound forms and soluble entities (sTNFR1 and sTNFR2). These soluble molecules circulate in serum and can bind TNF‐α, serving as natural antagonists. sTNFR1 and sTNFR2 are believed to regulate TNF‐α signaling during inflammatory responses and can act as DcRs (Figure 1) [58, 59].

In RA patients, the levels of both sTNFRs are elevated [60]. Although these receptors can act as DcRs, levels of sTNFRs in RA appear insufficient to prevent TNF‐α‐induced inflammation [60]. In another study, serum sTNFR levels were strong predictors of RA mortality [61].

SLE patients exhibit elevated sTNFR levels. Plasma sTNF‐RI levels were associated with disease severity and renal involvement in SLE patients [62].

sTNF‐R1 and sTNF‐R2 were detected at high levels in the CSF of MS patients [63]. There was a positive association between sTNFR1 and increased disability in MS patients [7]. Studies demonstrated that blocking sTNF improves functional outcomes in experimental autoimmune encephalomyelitis (EAE) and promotes axon preservation and remyelination [64, 65].

In MS, high levels of sTNFR1 were positively correlated with disease severity. In contrast, elevated levels of sTNFR2 were negatively correlated with severity. This suggests that these two receptors play different roles in the immunopathological mechanisms of MS [7]. sTNFR1 may contribute to neurodegeneration, while sTNFR2 may serve a protective role in remyelination [66, 67].

### 2.5. IL‐18BP

IL‐18BP is a soluble neutralizing protein that binds to IL‐18. IL‐18 promotes IFNγ and other proinflammatory cytokines during immune responses to pathogens or damage. Furthermore, IFNγ production induces IL‐18BP, which operates as a negative feedback mechanism (Figure 1) [68].

The dysregulation of IL‐18 and IL‐18BP is significantly associated with immune‐mediated diseases, especially those where IFNγ plays a pathological role, such as macrophage activation syndrome (MAS) [69]. MAS is a serious and potentially lethal complication associated with rheumatic diseases that typically occur within the framework of systemic juvenile idiopathic arthritis (sJIA). However, it can also manifest less frequently in SLE and Kawasaki disease [70].

Higher concentrations of IL‐18BP are observed in SLE patients [71]. These were higher in active disease than in remission. The high concentrations of IL‐18BP in the serum of active SLE patients indicate a potential role in the disease’s pathogenesis and progression [72].

In comparison with healthy controls, patients with pSS exhibit increased serum IL‐18BP levels, suggesting that IL‐18BP may play a role in the pathophysiology of pSS [73].

### 2.6. VEGFR‐1

VEGF‐A, commonly known as VEGF, is a vital regulator of endothelial dysfunction, capillary permeability, and the process of angiogenesis [74]. VEGF‐A binds to two tyrosine kinase receptors (TKs), VEGFR‐1 (Flt‐1) and VEGFR‐2 (KDR in humans) [75]. The binding affinity of VEGFR‐1 for VEGF‐A is significantly greater than that of VEGFR‐2, while the kinase activity of VEGFR‐1 is so weak that it can act as a DcR for VEGF [76]. In contrast to the other VEGFR genes, VEGFR‐1 expresses two varieties of mRNA: one that encodes a full‐length receptor and another that encodes a shorter soluble protein referred to as soluble VEGFR‐1 (sFlt‐1 or sVEGFR‐1) [77]. VEGFR‐1 is expressed in endothelial macrophages, facilitating macrophage function and contributing to the pathogenesis of inflammatory diseases (Figure 1) [77].

DcRs that function as scavenger receptors do more than just block ligands; they actively remove or sequester them, reducing their availability. Circulating sVEGFR‐1 binds to VEGF‐A. By sequestering VEGF ligands, these receptors prevent them from interacting with VEGFR‐2, which is why they are sometimes referred to as VEGF scavengers [78].

In RA patients, sVEGFR‐1 expression was increased and correlated with VEGF. Serum sVEGFR‐1 levels were higher in patients with OA with early and longstanding RA and those with self‐limiting arthritis than in nonarthritic controls [79]. The VEGFR1 genetic variant is linked to the activity level of RA [80].

In patients with SLE, higher serum levels of sVEGFR‐1 are positively associated with increased disease activity [81]. The sVEGFR‐1 concentration is the highest in active SLE compared to inactive disease and in healthy individuals [82].

### 2.7. Soluble IL‐6 Receptor (sIL‐6R)

IL‐6 is a multifunctional cytokine with pro‐ and anti‐inflammatory properties. IL‐6 induces intracellular signaling pathways after binding to its membrane‐bound receptor (IL‐6R) (Figure 1) [83].

In addition to the membrane‐bound form of IL‐6R, soluble isoforms of IL‐6R that lack the cytoplasmic domain and are transmembrane exist [84]. The sIL‐6R can bind IL‐6 and block its signaling [84]. The sIL‐6R can bind IL‐6 and block its signaling [5]. The release of sIL‐6R is believed to significantly influence acute and chronic inflammation. When neutrophils reach the site of inflammation, sIL‐6R is released, which recruits leukocytes by activating nearby endothelial cells and inducing the release of chemokine [85].

The levels of sIL‐6R in synovial fluid show significant correlations with indicators of chronic synovitis and the extent of joint damage in patients with RA [86], as well as with leucocyte infiltration [87].

Serum levels of sIL‐6R were significantly higher in MS patients compared to healthy controls and were correlated with the severity of the disease [88].

### 2.8. ACKRs

ACKRs are cell surface receptors that contain seven transmembrane domains. They are structurally similar to traditional chemokine G‐protein coupled receptors (GPCRs). ACKRs bind to specific chemokines with high affinity but do not signal through G‐proteins or facilitate cell migration [89]. Ligand‐receptor complexes are internalized by β‐arrestin‐dependent endocytosis [90]. The complexes are then transported to endosomes and, subsequently, to lysosomes, where the chemokines are degraded. The receptor is then returned to the plasma membrane for repeated scavenging [74, 91]. Some of the receptors in this group include the Duffy antigen receptor for chemokines (DARC), D6 (ACKR2 or CCBP2), CCX‐CKR, and CXCR7 (Figure 1) [92].

Human DARC binds many proinflammatory CC and CXC chemokines [93]. DARC was first identified in red blood cells but has also been found to be a prevalent receptor on vascular endothelial cells, which serve as the primary site for leukocyte transmigration in most tissues [94].

ACKR2 interacts with inflammatory CC chemokines (CCR1 to 5) but does not engage with homeostatic CC chemokines or CXC, CX, or CX3C chemokines [91]. ACKR2 is found in many organs, including barrier tissues like the skin, lungs, gut, and placenta. It is also expressed in populations of leukocytes, including T lymphocytes and their subsets, innate‐like B cells, and alveolar macrophages [95].

ACKR2 can recruit Tregs. The loss of ACKR2 may enhance Th17 polarization in specific models. Therefore, ACKR2 modulates the development of Th17 cells and Tregs [4, 96].

CXCR7 can bind to a ligand for CXCR3, CXCL11/I‐TAC, and a ligand for CXCR4, CXCL12/SDF‐1 [97]. Its expression has been found in T and B cell subsets, activated endothelial cells, fetal hepatocytes, the placenta, and vascular endothelium [97, 98].

In autoimmune diseases, the presence of ACKR2 on lymphatic endothelial cells decreases the accumulation of inflammatory chemokines near lymphatic capillaries. This reduction promotes fluid flow and facilitates the migration of activated dendritic cells (DCs) into neighboring lymph nodes, where they activate autoreactive T cells [99, 100].

DARC is upregulated in endothelial cells of CNS microvessels during EAE and in animal models of MS, and it contributes to EAE pathogenesis. Subcortical white matter micro‐vessel staining was positive for DARC in human MS brains compared to control tissue [101]. As a result, DARC plays a significant role in transporting inflammatory chemokines across the blood‐brain barrier during neuroinflammation and influences the severity and progression of the disease [101].

Research on MS indicates that ACKR2 mRNA levels in PBMCs from individuals with relapsing‐remitting MS (RRMS) are lower than those in healthy individuals [102].

In SLE, it has been indicated that ACKR2 expression increases in the kidneys and lungs of mice developing SLE. Additionally, ACKR2 inhibits T‐cell proliferation and the development of tertiary lymphoid structures, but it is not crucial for reducing autoimmune tissue damage in lupus‐susceptible B6lpr mice [95].

Chronic prostatitis and pelvic pain syndrome (CP/CPPS) is an autoimmune inflammatory disorder that leads to discomfort in the pelvic region or perineum, accompanied by the infiltration of inflammatory cells in the prostate [97]. Research on autoimmune prostate conditions shows that activating CXCR7 can reduce inflammation in chronic prostatitis by influencing the Th17/Treg cell ratio and affecting cell death in prostate epithelial cells. It also helps decrease fibrosis in chronic prostatitis [103].

RA, SLE, MS, IBD, psoriasis, T1D, MAS, DcR3, IL‐1RII, TRAIL receptors, sTNFRs, IL‐18BP, VEGFR‐1, ACKRs, FasL, and TL1A. The Figure 1 is an original figure created by the authors of the manuscript.

Unlike cytokines and chemokines, well‐defined DcRs for damage‐associated molecular patterns (DAMPs) are relatively uncommon. DAMPs, which consist of high‐mobility group box 1 (HMGB1), S100 proteins, and heat shock proteins (HSPs), are released from cells that are damaged or in the process of dying. They activate the innate immune system by interacting with pattern recognition receptors (PRRs), including toll‐like receptors (TLRs), and receptor for advanced glycation end products (RAGE) [104]. However, some functional analogs are available.

### 2.9. Soluble RAGE (sRAGE)

The RAGE is a multiligand PRR belonging to the immunoglobulin superfamily [105]. sRAGE is a form of RAGE that is truncated, missing the transmembrane and cytoplasmic domains, and is present in circulation either as a cleaved or alternatively spliced variant of RAGE, functioning as an extracellular DcR that inhibits RAGE by binding to its ligands (HMGB1 and S100) without triggering any cellular responses [106]. They do not internalize ligands, but instead scavenge DAMPs, preventing them from reaching cell‐surface RAGE [107].

Levels of sRAGE have been associated with autoimmune disorders. In RA and psoriasis, generally, lower levels of sRAGE are linked to disease activity, severity, inflammation, and increased vascular risk [108–110].

### 2.10. Soluble TLRs (sTLRs)

TLRs are components of the innate immune system specialized in recognizing common microbial motifs, or pathogen‐associated molecular patterns (PAMPs) [111]. Recently, a soluble form of TLR, released by proteolytic cleavage or alternative splicing, has been discovered and appears to have an inhibitory role [109]. This form binds to TLR ligands (HMGB1 and HSPs) without activating signaling pathways [112].

In SLE, the dysregulation of TLR signaling (between soluble and membrane forms) is thought to be involved [112].

### 2.11. CD24–Siglec‐10 Axis

CD24 is a small, heavily glycosylated protein that is anchored to the cell membrane by a glycosylphosphatidylinositol (GPI) link and acts as a checkpoint. It is expressed on a variety of cell types, including immature hematopoietic cells, B cells, T cells, DCs, and epithelial cells. The glycan side chains of CD24 contain sialic acid residues, which are essential for the protein’s interaction with Siglecs (sialic acid‐binding immunoglobulin‐like lectins) [113].

Siglec‐10 (human) is an inhibitory receptor found on myeloid cells, B cells, and DCs, featuring ITIM motifs (immunoreceptor tyrosine‐based inhibitory motifs) in their cytoplasmic regions [114].

DAMPs, such as HMGB1, HSP70, and HSP90, interact with the glycosylated surface of CD24. CD24 then presents these complexes to Siglec‐10 on immune cells, which sends an inhibitory signal into the immune cell, decreasing the production of inflammatory cytokines [106, 113].

Therefore, CD24 can be characterized as a noncanonical DcR, as it lacks intrinsic signaling capabilities but instead displays DAMPs to an inhibitory receptor (Siglec) to divert inflammatory activation. Mice lacking CD24 exhibit increased vulnerability to autoimmune diseases such as EAE and SLE [115].

## 3. Conclusion

DcRs become key regulators of immune homeostasis, modulating proinflammatory and anti‐inflammatory processes in autoimmune disorders. As cytokine molecular traps, these receptors, including DcR3, IL‐1RII, TRAIL receptors, sTNFRs, IL‐18BP, VEGFR‐1, and ACKRs, modulate immune cell activation, apoptosis, and inflammatory cascades. They have a dual role. Depending on the situation, DcRs can either inhibit or induce autoimmune disorders. Dysregulation of these receptors—whether through overexpression, deficiency, or dysfunctional ligand binding—is a characteristic of autoimmune diseases such as RA, SLE, IBD, MS, MAS, T1D, and psoriasis. This imbalance disrupts immune tolerance, prolongs chronic inflammation, and increases tissue damage. In summary, DcRs are not bystanders but key players in autoimmune disease. Their action will likely activate multiple pathways, and future studies of DcRs may lead to more effective therapies, rarer side effects, and enhanced life quality for patients with autoimmune diseases.

## Disclosure

No third‐party services or individuals outside the listed authors were involved in the research or manuscript preparation.

## Conflicts of Interest

The authors declare no conflicts of interest.

## Author Contributions

Hadiseh Farahani and Ali Ghazavi conceived the concept and designed the manuscript structure. Hadiseh Farahani, Ghasem Mosayebi, Ali Ganji, Ali Ghazavi, and Parviz Kokhaei collected and analyzed relevant literature and revised the paper. Hadiseh Farahani drafted the initial version of the manuscript and created the figure.

## Funding

No funding was received for this manuscript.

## Data Availability

The data that support the findings of this study are available on request from the corresponding author. The data are not publicly available because of privacy or ethical restrictions.
